# Cholesteatoma disease recidivism after canal wall up tympanomastoidectomy with or without obliteration (CLEAR-EAR): A protocol for a randomized controlled trial

**DOI:** 10.1371/journal.pone.0350772

**Published:** 2026-07-21

**Authors:** Chiara Erfurt, Maud J. de Rooij, Robert J. Stokroos, Louise V. Straatman, Hans G. X. M. Thomeer

**Affiliations:** 1 Department of Otorhinolaryngology and Head & Neck Surgery, University Medical Centre Utrecht, Utrecht, The Netherlands; 2 UMC Utrecht Brain Centre, Utrecht University, Utrecht, The Netherlands; Teikyo University Hospital Mizonokuchi, JAPAN

## Abstract

**Background and rationale:**

Cholesteatoma treatment relies on surgery with disease removal as the primary goal. While canal wall down (CWD) and canal wall up (CWU) tympanomastoidectomy remain standard approaches, mastoid obliteration has gained popularity. Canal wall up preserves ear canal anatomy and adding the obliteration technique suggests lower recurrence and residual rates. However, controversy persists and surgical consensus is lacking. Current decision-making relies on personal experience and patient-related factors rather than high-quality evidence. This study therefore investigates whether CWU with obliteration reduces cholesteatoma recurrence and residual disease compared to CWU without obliteration, by comparing recidivism rates for both interventions. Additionally, complications, hearing outcomes, quality of life and economic evaluation will be assessed. This study will assess long-term outcomes with a follow up of 5 years.

**Methods:**

This multicentre single-blind randomized controlled trial (RCT) will include 178 adults undergoing CWU tympanomastoidectomy for cholesteatoma removal, randomized in a 1:1 ratio to CWU with obliteration or without obliteration. Recurrent or residual disease is assessed by micro-otoscopy at 6–18 weeks and by micro-otoscopy and DW-MRI at one, three, and five years postoperatively. Suspected disease is confirmed during revision surgery when indicated. Secondary outcomes include hearing, complications, quality of life, and economic evaluation.

**Discussion:**

This is the first prospective RCT comparing CWU with and without obliteration. By establishing the follow-up period at five years, we aim to assess the true rates of recidivism, as literature indicates that most recurrent diseases are detected several years after surgery, with incidence appearing to rise approximately three years postoperatively. Key strengths include comparative hearing outcome assessment, quality of life evaluation, and economic evaluation. This multicentre trial aims to provide high-quality evidence to inform clinical guidelines and support evidence-based decision-making in cholesteatoma treatment.

**Trial registration:**

Netherlands Trial Register (CCMO): NL86362.041.24. Registered on 13 March 2025.

## Introduction

Cholesteatoma develops through retraction or ingrowth of the tympanic membrane’s skin into the middle ear, inducing chronic inflammation. Due to the inflammation and erosion of surrounding structures, a cholesteatoma leads to a constellation of symptoms such as hearing loss, tinnitus, vertigo, otalgia, otorrhea, aural fullness and taste disturbance. These problems severely impact not only physical health but also psychosocial wellbeing and quality of life. The only effective treatment is surgical removal. Without intervention, cholesteatoma can lead to severe complications such as meningitis, brain abscess, facial nerve paralysis, or permanent auditory or vestibular function loss.

The main goal of cholesteatoma treatment is to eradicate the disease and create a safe and dry ear, while preventing recurrence and preserving, or even optimizing, hearing. The optimal surgical approach has been debated for decades, with two traditional methods: canal wall down (CWD) and canal wall up (CWU) tympanomastoidectomy. Both have advantages and disadvantages, but the CWU technique is increasingly favoured. CWD surgery creates a radical cavity, allowing wide visual exposure for thorough removal, minimizing the risk of residual cholesteatoma. However, a radical cavity requires regular debridement, increases risk of recurrent infections, and makes the fitting of hearing aids more challenging. Conversely, the CWU approach preserves the ear canal’s normal anatomy, resulting in fewer inflammations, better hearing aids fitting, and no need for regular outpatient cleaning of the ear [1,2]. However, the major drawback of CWU is the reported higher risk of recurrent and residual disease. CWD tympanomastoidectomy shows recidivism rates (including both recurrent and residual disease) of 0–17%, whereas CWU rates range from 9% to 60% [3,4].

Over the past decade, there has been growing interest in reducing recidivism rates and improving postoperative hearing outcomes. One promising technique is obliteration of the mastoid and epitympanic area following either CWD or CWU tympanomastoidectomy. Obliteration involves filling the surgically created cavity with autologous bone dust (also referred to as bone pâté) or synthetic materials (like hydroxyapatite or Bonalive®/bioactive glass). The aim is to prevent the tympanic membrane retraction through obliteration, thereby reducing the risk of cholesteatoma recurrence or residual disease. Mercke was among the first otologic surgeons to report promising outcomes with obliteration combined with CWD tympanomastoidectomy [5].

When Mercke first presented his results, diffusion-weighted magnetic resonance imaging (DW-MRI) had not yet been developed, making postoperative cholesteatoma detection more difficult. At that time, all patients underwent a ‘second look’ operation, in which the ear was surgically reopened after a certain period to check for recurrence. Regarding obliteration, absence of appropriate imaging during cholesteatoma follow-up complicates the detection of residual disease and may also render its removal more challenging. The development of the DW-MRI addressed this concern by providing greater diagnostic assurance and is now routinely used for postoperative monitoring in some parts of the world, particularly in Europe [6,7]. With DW-MRI, the obliteration technique was reintroduced and extended to the CWU approach, as patients could be followed more reliably.

In their systematic review of the available retrospective studies, van der Toom et al. demonstrated that mastoid obliteration reduces recurrence rates compared to non-obliteration CWD and CWU techniques [8]. They reported an overall recurrent disease rate of 4.6% and an overall residual disease rate of 5.4% after mastoid obliteration, representing a substantial reduction compared to CWD (0–17%) and CWU (9–60%) techniques without obliteration [4]. This improvement can be attributed to the principles of mastoid obliteration, which eliminate the mastoid cavity as a potential site for disease recurrence. Combined with CWU tympanomastoidectomy it appears to be an effective strategy, whilst preserving the normal anatomy of the external auditory canal.

Currently, the decision to obliterate the mastoid cavity appears to depend largely on personal experience, preferences, and scientific beliefs. Within the current ENT community, the utility of obliteration remains widely discussed and continues to be a topic of interest in literature, at congresses, and within ENT societies. The superiority of the technique has been explored primarily through retrospective studies with numerous confounding factors and high risk of bias (including but not limited to differences in patient-related factors, surgical technique, obliteration material, follow-up periods, and exclusion/inclusion criteria). Furthermore, current research rarely categorizes outcomes based on cholesteatoma extension and localisation, as for example classified using the STAMCO [9].

Hearing is another important outcome measure. Most literature focuses on hearing outcomes after CWD or canal wall reconstruction surgery with mastoid and epitympanic obliteration and less on outcomes after CWU. Numerous studies demonstrate that these techniques preserve or even moderately improve preoperative hearing [1,10–13]. However, the literature on hearing outcomes following CWU with obliteration is limited. The few recent retrospective studies show promising results suggesting this approach also preserves hearing [14–16]. When obliterating the epitympanum, part of the ossicular chain (malleus head and incus) must be removed, which poses a risk for postoperative hearing loss. It is therefore important to investigate whether CWU tympanomastoidectomy with obliteration affects hearing outcomes, to better counsel patients on their expected postoperative hearing ability.

Changing clinical practice requires support from high quality prospective research. The aim of this randomized controlled trial is therefore, firstly, to determine whether CWU tympanomastoidectomy with obliteration reduces cholesteatoma recurrence and residual rates compared to CWU tympanomastoidectomy without obliteration. Secondly, hearing outcomes will be evaluated to investigate whether either technique yields better postoperative hearing. Thirdly, quality of life will be measured using a validated questionnaire and the cost-efficiency will be calculated.

## Methods

### Study design and setting

This study is a multicentre, two-arm randomized controlled trial conducted in both secondary care hospitals and tertiary academic centres. The trial will employ a single-blinded design, and a total of 178 participants will be enrolled. Participant recruitment is scheduled to commence in August 2026 and is expected to be completed within the subsequent 18 months. Eligible participants will be, after written informed consent, randomly assigned in a 1:1 allocation ratio to one of two intervention groups; the non-obliteration (A) or obliteration (B) group. Randomization will be centre-stratified and a 4,6,8 block randomization will be used. Measurements will be conducted at baseline, post-intervention (6–18 weeks), and at 1-, 3- and 5-year follow-up. The total study duration will be 7 years (84 months). The first 18 months will be dedicated to participant recruitment. Follow-up assessments will be completed over the subsequent 5 years. The final year will also be allocated to data analysis, interpretation of results, and reporting of the trial findings. The trial is expected to be completed in August 2033, with results anticipated by early 2034. Preliminary results will be disseminated prior to trial completion. The study protocol adheres the SPIRIT guidelines, and trial results will be reported using the CONSORT guidelines. This trial is registered in the Netherlands Trial Register (CCMO; NL86362.041.24).

### Eligibility criteria

Patients are considered eligible if they are scheduled to undergo a CWU tympanomastoidectomy based on clinical and/or radiological suspicion of cholesteatoma. Patients will only be included when the surgeon determines, based on the preoperative assessment, that a transcanal (full endoscopic) approach is not feasible and that a mastoidectomy is therefore necessary. Eligible patients must be at least 18 years of age, be willing and able to provide written informed consent prior to study participation, have sufficient understanding of written Dutch, French, or English, and be medically fit to undergo general anaesthesia and surgical removal of cholesteatoma.

Patients will be excluded in cases of revision surgery for residual disease in the presence of a normal, intact or reconstructed tympanic membrane; in patients who have previously undergone mastoid obliteration; in case of congenital cholesteatoma; when the indication for surgery is other than cholesteatoma; when the extent of disease is such that obliteration is deemed unavoidable; when life expectancy is less than five years; in the presence of comorbidities or disorders that could interfere with completion of study questionnaires; in case of a compromising anatomical situation; or when there is a contraindication to undergo DW-MRI.

### Sample size

To detect a clinically and statistically relevant difference in the primary outcome (residual or recurrent disease), a minimum sample size of 160 participants is required. The sample size calculation was based on retrospective data from University Medical Centre Utrecht (20.3% in obliteration group and 45.7% in non-obliteration group, January 2015-March 2020) and rates reported in our recent systematic review of the literature, representing the most comparable dataset currently available. For sample size calculation, conservative rounded estimates of 20% versus 40% were used. A clinically relevant difference of 20% was defined as the target effect size, as this magnitude would substantially impact surgical decision-making and patient counselling regarding revision surgery risk and long-term disease control.

The calculation was performed using a two-independent proportions power analysis with a Z test with pooled variance, with a two-sided alpha of 0.05 and a power of 0.80. All assumptions used in the sample size calculation are summarized in S1 Table. The sample size calculation (PASS version 2008) resulted in 80 patients per study arm (160 participants in total). To compensate for potential dropouts, a 10% margin is implemented, resulting in a total sample size of 178 participants.

### Recruitment

This study is designed as a multicentre trial, with seven centres participating in participant recruitment and performing the surgical procedures. The target enrolment rate is one to two participants per centre per month, resulting in a total sample of 178 participants over an 18-month recruitment period across all centres. The participating sites are located in both the Netherlands and Belgium. The four Dutch centres include the University Medical Centre Utrecht, Amsterdam University Medical Centre, Deventer Hospital, and Sint Antonius Hospital. The three Belgian centres include University Hospitals Leuven, Cliniques Universitaires Saint-Luc Brussels, and Centre Hospitalier Universitaire UCL Namur. Collaborating otologists will screen and include eligible participants with an indication for a CWU tympanomastoidectomy, regardless of whether this concerns primary or revision surgery, provided all other eligibility criteria are met. Recruitment is scheduled to commence in August 2026.

### Randomization and blinding

Eligible participants (after providing written informed consent) will be randomized to either the obliteration or non-obliteration group using a web-based randomisation tool in Castor EDC. Patients are randomized into one of two groups in an equal 1:1 allocation ratio, using variable block sizes of 4, 6, and 8. The randomization sequence will be generated automatically by the Castor EDC system. Stratification will be used to distribute the two groups as evenly as possible between the participating centres. This study uses single-blind allocation, in which participants are blinded to the treatment received. Blinding is considered necessary because quality of life will be a key subjective outcome measure. The treatment allocation (obliteration or non-obliteration) will not be reported in the surgical report or in the letter to the general practitioner. Allocation will only be recorded in Castor EDC and in the Investigator Site File. Blinding will only be lifted if this is considered necessary for participants’ safety. The participant and/or general practitioner may request unblinding through the otologist who performed the surgery. The treating physician will determine whether unblinding is justified and will inform the researcher if this occurs. After completion of the final quality of life questionnaire (at 36 months), the treatment allocation will be recorded in the electronic patient record. Treating physicians, radiologists, and audiologists are not blinded, as outcome assessments are integrated into standard clinical care.

### Intervention

All subjects with clinical and/or radiological suspicion of cholesteatoma will undergo a CWU surgical approach to remove the cholesteatoma with the aim of creating a dry and safe ear. Half of the cohort (group B, N = 89) will undergo CWU with mastoid obliteration. Obliteration may be performed using various materials: autologous bone dust, cartilage, muscle, synthetic material (Bonalive® or hydroxyapatite), or a combination of these. The study evaluates the outcomes of the two surgical strategies (with or without obliteration). No products will be studied.

### Assessments and outcomes

**Fig 1:**
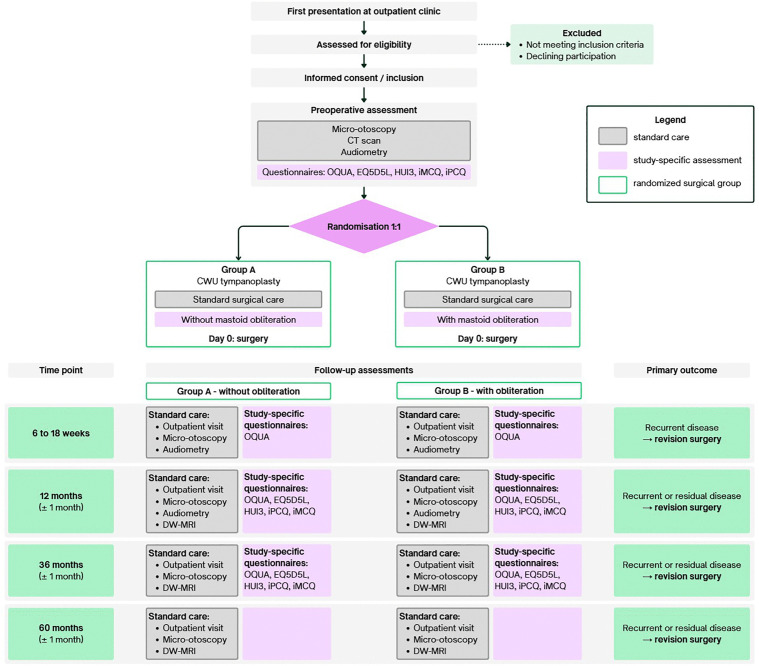
Study flowchart and visit schedule for the CLEAR-EAR trial.

All participants will undergo clinical assessments at baseline, postoperatively (6–18 weeks), and at 1-, 3- and 5-year follow-up. An overview of the trial including the visit schedule can be found in Fig 1.

At baseline, the preoperative assessment includes physical examination of the ear (micro-otoscopy), audiometry and CT imaging. These investigations are all part of standard care in both the Netherlands and Belgium. In addition, patient-reported outcome measures (PROMs) assessing quality of life and economic outcomes will be collected as part of the trial.

The first postoperative assessment will take place at 6–18 weeks and will include micro-otoscopy and audiometry (standard care), as well as PROMs assessing quality of life. Subsequent follow-up visits will take place at approximately 1, 3 and 5 years after surgery. During these visits, micro-otoscopy and DW-MRI will be performed as part of standard care. PROMs related to quality of life and economic outcomes will again be collected.

In accordance with standard clinical practice, revision surgery will be performed if there is suspicion of recurrent or residual pathology, based on either clinical (micro-otoscopy) or radiological (DW-MRI) findings. If revision surgery confirms recurrent or residual cholesteatoma, the type of recidivism and future questionnaires will be recorded. If revision surgery does not confirm disease (false-positive suspicion), the participant will continue clinical follow-up as planned, unless reconstructive surgery aimed at hearing improvement has been performed.

During the first three years of follow-up, patients should not undergo another type of ear surgery. If this is considered unavoidable by the treating physician, this will be documented as a protocol deviation, but the participant will remain in the study and continue follow-up as planned.

#### Baseline characteristics.

Baseline characteristics will include patient demographics (age and gender), date of first presentation at the outpatient clinic, date of surgery, operated side (right/left), and antibiotic treatment within two weeks prior to surgery. Comorbidities and lifestyle factors will be recorded, including smoking status, obesity, asthma, diabetes mellitus, chronic obstructive pulmonary disease (COPD), allergies, and rhinosinusitis. In addition, the number and type of previous ear surgeries (not listed in the exclusion criteria) will be documented. Baseline characteristics will be reported descriptively for each treatment group. No subgroup analyses based on demographic characteristics are planned.

#### Cholesteatoma recidivism.

The primary outcome measure is confirmed recurrent or residual cholesteatoma at five years after index surgery. This is defined as a composite binary endpoint where patients are classified as having recidivism if either recurrent or residual disease is confirmed at the 60-month assessment. Key secondary endpoints include recurrence status at 12 and 36 months.

Follow-up assessments including micro-otoscopy and DW-MRI will be performed as described (Fig 1). A newly formed retraction pocket from the tympanic membrane correlates with recurrent disease (clinical diagnosis), whereas cholesteatoma formation behind a normal or an intact tympanic membrane after primary surgery correlates with residual disease (radiologic diagnosis). Both will be confirmed by revision surgery.

#### Hearing outcomes.

Hearing outcomes will be assessed by audiometric testing, including pure-tone average (PTA) thresholds calculated over 0.5, 1, 2 and 4 kHz for air conduction (AC) and bone conduction (BC), and by speech audiometry. The preoperative audiogram should not have been measured more than 6 months prior to surgery. Follow-up assessments will be performed at 6–18 weeks and at approximately 1 year postoperatively (Fig 1).

#### Surgical characteristics.

Surgical characteristics will be documented for all procedures. The localization and extension of the cholesteatoma will be described using the STAMCO classification [9]. This classification also includes the presence of preoperative complications and the ossicular chain status prior to removal of the cholesteatoma. Differences in STAMCO classification between the two randomized groups will be evaluated and described.

Additional intraoperative characteristics will be recorded, including the material used for mastoid obliteration (e.g., Bonalive®, bone dust, hydroxyapatite, cartilage, muscle) as documented in the surgical report. Ossicular chain reconstruction (OCR) will also be recorded. If OCR is performed, the reconstruction type (e.g., PORP, TORP, incus interposition, or type III tympanoplasty) will be documented. Operative time will be recorded in minutes for each procedure.

For any revision surgeries performed during the follow-up period, intraoperative findings and procedural details will be collected from the corresponding surgical reports.

#### Quality of life.

Quality of life will be assessed using a validated PROM in the form of an otological questionnaire (Otology Questionnaire Amsterdam; OQUA) [17]. This instrument consists of 34 items covering eight domains of ear complaints and evaluates both the frequency and severity of symptoms as well as the impact on quality of life.

#### Complications.

Complications related to cholesteatoma will be recorded (e.g., labyrinth fistula, dural defect, meningoencephalocele, dehiscence of facial nerve, interrupted bony tegmen plate). In addition, surgical complications will be documented, including wound infection, bleeding, inner ear damage (resulting in perceptive hearing loss or dizziness), facial nerve damage causing facial paralysis, chorda tympani damage resulting in taste disturbance, intracranial complications (e.g., tegmen damage or liquor leakage), and tinnitus. Only complications that occur within the first 6 weeks after surgery will be registered, as these are attributable to the surgical procedure.

#### Health economic evaluation.

A cost-utility analysis will be performed from a societal perspective with a lifetime horizon, following the Dutch guideline for economic evaluations in healthcare. Alongside the trial, data will be collected and extrapolated using a decision-analytical model. Healthcare resource use will be measured with the iMCQ and productivity losses with the iPCQ. Surgical costs will be calculated using a bottom-up approach, and health-related quality of life will be assessed with the HUI-3, validated against the EQ-5D-5L. In a base case analysis Dutch unit costs will be applied, as the Belgian unit costs and a healthcare perspective will be applied in a scenario analysis. Extensive sensitivity analyses including deterministic, probabilistic, scenario and value of information (VOI) analyses will be conducted. The results of the cost-effectiveness analysis will be combined with observed number eligible patients to estimate the budget impact.

### Data management and data analysis

All study procedures and tests are part of standard clinical care, the only additional elements are the informed consent process, randomization, and the collection of study questionnaires. Baseline characteristics, preoperative computed tomography (CT) scans, postoperative DW-MRI scans, and pre- and postoperative audiometric data will be extracted from the electronic patient records. Questionnaires, operative reports, and randomization will be performed and collected using the Castor EDC platform. All study data will be stored in a clinical research file, which will be added to the Castor database. Medical images will be transferred (in pseudonymized form) to a research imaging archive at the sponsor site.

Data handling and protection will be conducted in accordance with applicable laws and regulations, including GCP, 21 CFR Part 11, GDPR, and ISO 27001/9001 standards. Participant confidentiality will be maintained at all times, and individual data will not be disclosed to third parties. Each participant will receive a unique study number to protect their privacy. The original informed consent forms will be stored securely at the local site, while a copy will be added to the research file in Castor. The investigator will safeguard the key linking participants’ personal data to their study number, and access to the source data will be restricted.

The clinical database will be supplemented with outcomes from the validated OQUA questionnaire, which will be automatically distributed via email links through Castor at predefined timepoints. Email addresses will be entered in an encrypted manner, traceably only to the participant’s site of origin. Only the study team will have access to the database files in Castor. All generated data and metadata will be stored in a secure research folder structure with access control at each participating centre, and only pseudonymized data will leave the site of origin. The database will be maintained for 15 years after the study completion.

#### Statistical analyses.

All data analyses will be performed using SPSS v30.0 (SPSS Inc., IBM, USA) by the study biostatistician, with a significance level of p < 0.05. Missing data will be handled using multiple imputation by chained equations (MICE), generating 20 datasets pooled via Rubin’s rules, assuming missing at random. Effect sizes will be reported with 95% confidence intervals. Data will be presented according to the CONSORT Statement. Baseline characteristics will be compared between groups. The primary outcome of recidivism (recurrent and residual disease) at 60 months will be analysed using Chi-square or Fisher’s exact tests, with relative risk and absolute risk difference calculated. The primary analysis will be adjusted for site, surgeon experience, and STAMCO stage using logistic regression. Secondary time-to-event analyses will include Kaplan-Meier survival curves for recurrence-free survival, log-rank test stratified by centre, and Cox proportional hazards regression adjusted for centre and baseline prognostic factors (age, cholesteatoma extent, revision surgery). Recidivism rates at 12 and 36 months will be reported as key secondary outcomes using Chi-square or Fisher’s exact tests.

As exploratory analyses, within the STAM classification subgroups (STAM 1,2,3), Chi-square tests will be used to assess differences in recidivism. Hearing outcomes will be analysed using a mixed model for repeated measures (MMRM), with mean differences in air-bone gap between pre- and postoperative audiograms compared between groups. The groups will be subdivided according to ossicular chain status at the start of surgery, as classified by the STAMCO classification. Postoperative ossicular chain status will be additionally reported stratified by type of ossicular reconstruction performed. Hearing results will be visualized using the Amsterdam Hearing Evaluation Plot (AHEP) and scatterplots for speech audiograms.

The OQUA questionnaire data will be analysed using MMRM, with the primary inference based on the estimated mean difference at 36 months. Baseline characteristics, complications, and surgical details will be described using appropriate descriptive statistics. A cost-utility analysis will be performed using a base case analysis, extended by a scenario analysis as described.

An intention-to-treat (ITT) analysis approach will be used for all analyses, including all randomized participants analysed according to their allocated treatment group, regardless of protocol adherence or deviations. Dropout rates will be monitored, and any differential attrition between treatment arms will be accounted for in the statistical analysis using multiple imputation. A per-protocol (PP) sensitivity analysis will be conducted, excluding patients with major protocol deviations. Comparison of ITT and PP results will assess the robustness of findings to protocol adherence.

### Ethics and monitoring

Ethical approval was obtained from the Medical Research Ethics Committee NedMec (MREC NedMec; approval number: 24–344/G; 13 March 2025), confirming compliance with the Dutch Medical Research Involving Human Subjects Act (WMO) and applicable Dutch and European regulations. Written informed consent will be obtained by the treating physician from all participants prior to study participation.

Since the canal wall up procedure with or without obliteration is part of standard care for cholesteatoma patients, participants will not be exposed to additional risks beyond standard treatment. The research risk is therefore considered negligible.

Independent qualified monitors will conduct monitoring for all participating centres. The sponsor’s monitoring plan will be mirrored across all sites to ensure consistent quality assurance.

Adverse events (surgical complications within six weeks post-surgery) will be documented and reviewed quarterly by the investigators. Any serious adverse events (SAEs) are expected to be related to standard treatment rather than the study intervention. SAEs will only be reported if they occur during surgery or within six weeks postoperatively. The investigator will report all SAEs to the sponsor without undue delay via secured email. The sponsor will report SAEs to the accredited MREC through *ToetsingOnline* within 7 days for life-threatening events or deaths (with completion within 15 days) and within 15 days for all other SAEs.

The sponsor maintains liability insurance in accordance with Article 7 of the WMO. Protocol amendments will be reported to all relevant parties according to applicable regulations.

## Discussion

The mainstay of cholesteatoma treatment is surgery with effective removal of disease as the primary goal to achieve a safe and dry ear. As recurrent and residual disease rates remain high, many emerging surgical techniques are being explored and adopted by the international ENT community. One such technique is mastoid obliteration in CWU surgery, which may be superior to CWU tympanomastoidectomy without obliteration in reducing recidivism.

### Current evidence

Currently available data on mastoid obliteration is solely retrospective in nature, carrying a high risk of bias and selection effects. Multiple systematic reviews have highlighted the methodological limitations of existing studies. Van der Toom et al. systematically reviewed 13 studies on mastoid obliteration (both CWU and CWD approach) and found that all were retrospective cohorts, with prospective data collection in only three studies and no studies with prospectively calculated sample sizes [8]. The most common limitations identified were small patient numbers, short follow-up periods, inadequate follow-up methods, and lack of differentiation between recurrent and residual disease. Similarly, Illés et al. concluded in their meta-analysis of 11 studies that while mastoid obliteration significantly reduces combined residual and recurrent cholesteatoma rates, the quality of available data remains low [18]. Salem et al. explicitly called for randomized controlled trials to address the inherent selection bias in retrospective comparisons [19].

Despite these methodological limitations, the largest retrospective studies have reported promising results. Van der Toom et al. reported on 337 adult patients and found a combined recurrent and residual cholesteatoma rate of 7.6% in the obliteration group versus 34.9% in the CWU without obliteration group after five years of follow-up [20]. Similarly, Angeli et al. compared 123 patients and demonstrated five-year cholesteatoma-free survival of 91% with obliteration versus 63% without obliteration [21]. While these studies consistently suggest benefits of obliteration, they cannot adequately control for confounders such as cholesteatoma extent, surgical expertise, patient selection criteria, and evolving surgical techniques over time. Furthermore, as obliteration is a relatively new technique, most retrospective studies report shorter follow-up periods for the obliteration group. While short-term data may suggest lower recurrence rates, the limited long-term data available indicates that this difference may diminish over time, highlighting the need for extended follow-up in prospective studies. Additionally, existing studies provide limited data on comparative hearing outcomes, quality of life, and health economic implications, all critical factors for clinical decision-making.

### Strengths and innovation of this trial

This trial is the first prospective, randomized controlled trial evaluating whether CWU with obliteration reduces cholesteatoma recurrence and residual disease rates compared to CWU without obliteration. The randomized design eliminates selection bias and ensures balanced distribution of known and unknown confounders between treatment groups, addressing the fundamental limitation of all existing evidence. Unlike retrospective studies where surgeon preference and experience influence treatment allocation, randomization ensures that any observed differences in outcomes can be attributed to the surgical technique itself.

A key methodological strength is the comprehensive assessment of multiple clinically relevant outcomes. While retrospective studies have primarily focused on recidivism rates, this trial will systematically compare hearing outcomes. Quality of life will be assessed using validated instruments, providing patient-centred outcome data that is largely absent from existing literature. The health economic evaluation will determine cost-effectiveness by accounting for surgical time, complications, revision surgery rates, and long-term follow-up costs. The multidimensional approach reflects the complexity of surgical decision-making, where recidivism rates alone do not capture the full clinical picture.

The five-year follow-up period will enable accurate detection of recidivism with equal observation time for both groups. This addresses a critical limitation of retrospective studies where obliteration groups often have systematically shorter follow-up due to the technique’s relatively recent adoption. The standardized procedures will enhance validity and reproducibility of results. The multicentre, international design ensures applicability across different surgical practices and healthcare systems, improving generalizability beyond single-institution experiences.

### Limitations

Several limitations warrant consideration. First, patients with comorbidities interfering with questionnaire completion will be excluded, this may limit generalizability to the broader patient population. However, we anticipate that the included population will remain representative of most patients eligible for mastoid obliteration.

Second, involvement of multiple surgeons may introduce variability in surgical technique despite standardized procedural guidelines. However, this multi-surgeon approach enhances external validity and real-world applicability of our findings.

Third, different obliteration materials are permitted and will not be analysed separately. We do not expect this to impact outcomes, as the underlying pathophysiological mechanism of obliteration remains consistent across materials. Should unexpected heterogeneity arise, post-hoc subgroup analyses could be considered.

Fourth, our sample size calculation was based on retrospective data, which may have overestimated the true effect size. However, we consider our study adequately powered for clinically meaningful differences that would substantially impact surgical decision-making.

Nevertheless, this prospective randomized trial addresses critical gaps in the current evidence base.

### Clinical implications

The results of this trial will have important clinical implications. If mastoid obliteration proves superior in reducing recurrence and residual disease rates without compromising hearing outcomes, it could become the preferred surgical approach for cholesteatoma treatment. Conversely, if no significant difference is found, the additional surgical time and potential complications associated with obliteration may not be justified, particularly in resource-limited settings. The health economic evaluation will further inform clinical decision-making by providing evidence on cost-effectiveness. Ultimately, this study will generate the high-quality evidence needed to inform clinical guidelines and support evidence-based surgical practice for cholesteatoma treatment.

### Trial status

Protocol version 1.2 dated 25 March 2026. Recruitment is expected to initiate on first of August 2026 and complete on first of February 2028. Approximate date of trial completion: August 2033.

## Supporting information

S1 Table Assumptions table. ᵃ UMCU cohort, January 2015 to March 2020. ᵇ Magnitude considered to substantially impact surgical decision-making.
(DOCX)

S2 File SPIRIT 2025 checklist. (PDF)

S3 File Research protocol NL86362.041.24 v1.2. (PDF)

## Abbreviations

ENT: Ear, Nose, Throat
CWD: canal wall down
CWU: canal wall up
DW-MRI: diffusion-weighted magnetic resonance imaging
OQUA: otology questionnaire Amsterdam
PROM: patient-reported outcome measure
PTA: pure-tone average
AC: air conduction
BC: bone conduction
OCR: ossicular chain reconstruction
PORP: partial ossicular replacement prothesis
TORP: total ossicular replacement prothesis
iMCQ: medical consumption questionnaire
iPCQ: productivity cost questionnaire
HUI-3: health utilities index questionnaire
EQ-5D-5L: health questionnaire
CT: computed tomography
MMRM: mixed models for repeated measures
AHEP: Amsterdam hearing evaluation plot
SAE: severe adverse event
