# Supplementary material for: Cholesteatoma disease recidivism after canal wall up tympanomastoidectomy with or without obliteration (CLEAR-EAR): A protocol for a randomized controlled trial
Source: PLoS One. 2026 Jul 21;21(7):e0350772. doi: 10.1371/journal.pone.0350772 (PMC13387520; doi:10.1371/journal.pone.0350772)
Supplement: S1 Table — ᵃ UMCU cohort, January 2015 to March 2020. ᵇ Magnitude considered to substantially impact surgical decision-making. (DOCX) [file pone.0350772.s001.docx]

**S1 Table. Assumptions for sample size calculation**

| **Parameter** | **Value** | **Source/Justification** |
| --- | --- | --- |
| Expected recurrence rate (CWU without obliteration) | 40% | Conservative estimate (UMCU 45.7%)^a^ |
| Expected recurrence rate (CWU with obliteration) | 20% | Based on UMCU data (20.3%)^a^ |
| Target absolute difference | 20% | Clinically relevant treshold^b^ |
| Significance level (α) | 0.05 (two-sided) |  |
| Statistical power (1-β) | 0.80 |  |
| Statistical test | Two-proportion Z-test (pooled variance) |  |
| Allocation ratio | 1:1 |  |
| Required sample size per group | 80 |  |
| Anticipated dropout rate | 10% |  |
| Total required sample size | 178 |  |

ᵃ UMCU cohort, January 2015 to March 2020

ᵇ Magnitude considered to substantially impact surgical decision-making
