# Supplementary material for: Cholesteatoma disease recidivism after canal wall up tympanomastoidectomy with or without obliteration (CLEAR-EAR): A protocol for a randomized controlled trial
Source: PLoS One. 2026 Jul 21;21(7):e0350772. doi: 10.1371/journal.pone.0350772 (PMC13387520; doi:10.1371/journal.pone.0350772)
Supplement: S3 File — (PDF) [file pone.0350772.s003.pdf]

Cholesteatoma disease recidivism after canal wall up tympanomastoidectomy with or without obliteration: a randomized controlled trial.

PROTOCOL TITLE 'Cholesteatoma disease recidivism after canal wall up tympanomastoidectomy with or without obliteration: a randomized controlled trial **(CLEAR-EAR)**.'

**Protocol ID**	24U-0116
**Short title**	**CLEAR-EAR**<br>**C**ho**LE**steatom**A** **R**ecidivism – **E**valuation **A**nd **R**ecommendations
**Version**	1.2
**Date**	25-03-2026
**Principal investigator(s) and coordinating investigator (in Dutch: hoofdonderzoeker/ uitvoerder) en coördinerend onderzoeker**<br><br>**Multicenter research: per site**	**UMC Utrecht:**<br>**Dr. H.G.X.M. (Hans) Thomeer**<br>Department of Otorhinolaryngology and Head & Neck Surgery, University Medical Center Utrecht<br>Heidelberglaan 100, 3584 CX Utrecht, the Netherlands<br>E-mail: h.g.x.m.thomeer@umcutrecht.nl<br><br>**Multicenter:**<br>1. **UZ Leuven:** prof. dr. N. (Nicolas) Verhaert nicolas.verhaert@uzleuven.be<br>2. **Amsterdam UMC:** dr. T.P.M. (Thadé) Goderie t.goderie@amsterdamumc.nl<br>3. **Deventer Ziekenhuis (DZ):** dr. P.M.W. (Pauline) van Kempen p.kempenvan@dz.nl<br>4. **St. Antonius Ziekenhuis:** drs. D. (David) Kupperman d.kupperman@antoniusziekenhuis.nl<br>5. **Cliniques universitaires Saint-luc (bruxelles) :** prof. Dr. V. Topsakal vedat.topsakal@saintluc.uclouvain.be<br>6. **CHU UCL Namur :** dr. Jean-Philippe vanDamme jean-philippe.vandamme@chuuclnamur.uclouvain.be

**Other investigator(s)**	**Dr. L.V. (Louise) Straatman**<br>Department of Otorhinolaryngology and Head & Neck Surgery, University Medical Center Utrecht<br>Heidelberglaan 100, 3584 CX Utrecht, the Netherlands<br>E-mail: L.V.Straatman@umcutrecht.nl<br><br>**Prof. dr. R.J. (Robert) Stokroos**<br>Head of the department of Otorhinolaryngology and Head & Neck Surgery of the University Medical Center Utrecht<br>E-mail: r.j.stokroos@umcutrecht.nl

	**Dr. A.L. (Diane) Smit**<br>Department of Otorhinolaryngology and Head & Neck Surgery, University Medical Center Utrecht<br>Heidelberglaan 100, 3584 CX Utrecht, the Netherlands<br>E-mail: a.l.smit-9@umcutrecht.nl<br><br>**Drs. C. (Chiara) Erfurt**<br>Department of Otorhinolaryngology and Head & Neck Surgery, University Medical Center Utrecht<br>Heidelberglaan 100, 3584 CX Utrecht, the Netherlands<br>E-mail: c.erfurt@umcutrecht.nl<br><br>**Drs. M.J. (Maud) de Rooij**<br>Department of Otorhinolaryngology and Head & Neck Surgery, University Medical Center Utrecht<br>Heidelberglaan 100, 3584 CX Utrecht, the Netherlands<br>Email: m.j.derooij-19@umcutrecht.nl
**Sponsor (in Dutch: verrichter/opdrachtgever)**	Universitair Medisch Centrum (UMC) Utrecht
**Subsidising party**	UMC Utrecht
**Independent expert (s)**	**Dr. A.J.N. Bittermann**<br>A.J.N.Bittermann@umcutrecht.nl<br>Department of Otorhinolaryngology and Head and Neck surgery, University Medical Center Utrecht

TABLE OF CONTENTS

1. INTRODUCTION AND RATIONALE ... 9
2. OBJECTIVES ... 12
3. STUDY DESIGN ... 12
4. STUDY POPULATION ... 15
   4.1 Population (base) ... 15
   4.2 Inclusion criteria ... 15
   4.3 Exclusion criteria ... 15
   4.4 Sample size calculation ... 16
5. TREATMENT OF SUBJECTS ... 16
   5.1 Investigational product/treatment ... 16
6. METHODS ... 17
   6.1 Study parameters/endpoints ... 17
      6.1.1 Main study parameter/endpoint ... 17
      6.1.2 Secondary study parameters/endpoints ... 17
   6.2 Randomisation, blinding and treatment allocation ... 20
   6.3 Study procedures ... 21
   6.4 Withdrawal of individual subjects ... 22
   6.5 Replacement of individual subjects after withdrawal ... 22
   6.6 Follow-up of subjects withdrawn from treatment ... 22
   6.7 Premature termination of the study ... 22
7. SAFETY REPORTING ... 22
   7.1 Temporary halt for reasons of subject safety ... 22
   7.2 AEs, SAEs and SUSARs ... 22
      7.2.1 Adverse events (AEs) ... 22
      7.2.2 Serious adverse events (SAEs) ... 23
8. STATISTICAL ANALYSIS ... 24
   8.1 Primary study parameter(s) ... 24
   8.2 Secondary study parameter(s) ... 24
   8.3 Interim analysis (if applicable) ... 26
9. ETHICAL CONSIDERATIONS ... 26
   9.1 Regulation statement ... 26
   9.2 Recruitment and consent ... 26
   9.3 Benefits and risks assessment, group relatedness ... 27
   9.4 Compensation for injury ... 27
10. ADMINISTRATIVE ASPECTS, MONITORING AND PUBLICATION ... 27
   10.1 Handling and storage of data and documents ... 27
   10.2 Monitoring and Quality Assurance ... 28
   10.3 Amendments ... 29
   10.4 Annual progress report ... 29
   10.5 Temporary halt and (prematurely) end of study report ... 29
   10.6 Public disclosure and publication policy ... 29
11. REFERENCES ... 30

LIST OF ABBREVIATIONS AND RELEVANT DEFINITIONS

**ABR**	**General Assessment and Registration form (ABR form), the application form that is required for submission to the accredited Ethics Committee; in Dutch: Algemeen Beoordelings- en Registratieformulier (ABR-formulier)**
**AE**	**Adverse Event**
**AHEP**	**Amsterdam Hearing Evaluation Plot**
**AR**	**Adverse Reaction**
**CA**	**Competent Authority**
**CCMO**	**Central Committee on Research Involving Human Subjects; in Dutch: Centrale Commissie Mensgebonden Onderzoek**
**CLEAR-EAR**	**ChoLEsteatomA Recidivism – Evaluation And Recommendations**
**CV**	**Curriculum Vitae**
**CWD**	**Canal wall down**
**CWU**	**Canal wall up**
**DSMB**	**Data Safety Monitoring Board**
**DW-MRI**	**Diffusion-weighted magnetic resonance imaging**
**EU**	**European Union**
**EudraCT**	**European drug regulatory affairs Clinical Trials**
**ENT**	**Ear-Nose-Throat**
**EQ-5D-5L**	**EuroQol-5 Dimensions**
**GCP**	**Good Clinical Practice**
**GDPR**	**General Data Protection Regulation; in Dutch: Algemene Verordening Gegevensbescherming (AVG)**
**HUI3**	**Health Utility Index - 3**
**IB**	**Investigator's Brochure**
**IC**	**Informed Consent**
**iMCQ**	**Medical Consumption Questionnaire**
**IMP**	**Investigational Medicinal Product**
**IMPD**	**Investigational Medicinal Product Dossier**
**iPCQ**	**Productivity Cost Questionnaire**
**ITT**	**Intention-to-treat analysis**
**METC**	**Medical research ethics committee (MREC); in Dutch: medisch-ethische toetsingscommissie (METC)**
**OQUA**	**Otology Questionnaire Amsterdam**

**PROM**	**Patient-reported outcome measure**
**PSA**	**Probabilistic sensitivity analysis**
**Recidivism**	**Total number/rate of residual and recurrent disease**
**(S)AE**	**(Serious) Adverse Event**
**SPC**	**Summary of Product Characteristics; in Dutch: officiële productinformatie IB1-tekst**
**Sponsor**	**The sponsor is the party that commissions the organisation or performance of the research, for example a pharmaceutical company, academic hospital, scientific organisation or investigator. A party that provides funding for a study but does not commission it is not regarded as the sponsor, but referred to as a subsidising party.**
**SUSAR**	**Suspected Unexpected Serious Adverse Reaction**
**UAVG**	**Dutch Act on Implementation of the General Data Protection Regulation; in Dutch: Uitvoeringswet AVG**
**WMO**	**Medical Research Involving Human Subjects Act; in Dutch: Wet Medisch-wetenschappelijk Onderzoek met Mensen**

SUMMARY

Rationale: The mainstay of cholesteatoma treatment is surgery with effective and safe removal of the disease as the principal goal. New techniques find their way in the international otologic community by proving lower recurrent and residual disease rates compared to the conventional techniques. One newly implemented and previously described technique is the obliteration technique of the mastoid. Although this approach has become more popular in recent years, high-quality evidence is missing.

Objective: The primary aim of this study is to investigate whether a canal wall up (CWU) tympanomastoidectomy with obliteration of the mastoid and paratympanic or epitympanic spaces reduces cholesteatoma recurrence and residual rates compared to the same approach without obliteration. Secondarily, hearing outcomes after both surgical techniques are compared to investigate whether one of the two results in superior postoperative hearing. The quality of life will be measured using the Otology Questionnaire Amsterdam (OQUA). The economic evaluation will involve collecting the EQ-5D-5L, HUI-3, iPCQ and iMCQ questionnaires as well as direct intervention costs from the sponsoring centre.

Study design: The proposed study is a single-blind randomized controlled trial. One hundred seventy-eight patients will be included. Patients are randomized into one of two groups in an equal 1:1 allocation ratio. The randomization will be centre-stratified and a 4,6,8 block randomization will be used.

Study population: Patients above the age of 18 who undergo a CWU approach for removal of cholesteatoma in one of the participating centres.

Intervention (if applicable): Group A will undergo a CWU approach without obliteration and group B a CWU approach with obliteration.

Main study parameters/endpoints: Rates of recurrent or residual cholesteatoma are evaluated by diffusion-weighted magnetic resonance imaging (DW-MRI) and micro-otoscopy after approximately one, three and five years. If diffusion restriction is seen on DW-MRI and/or retraction of the tympanic membrane is seen during micro-otoscopy, suspicion for recurrent or residual disease will be recorded. To confirm the diagnosis and to determine whether the cholesteatoma is either recurrent or residual, we will analyse the tympanic membrane description at the beginning of the revision surgery. All other surgical outcome measures are recorded in the surgical report. Pure tone audiograms, including air and bone conduction, and speech recognition scores are evaluated pre- and postoperatively for each patient. The EQ-5D-5L, HUI-3, iMCQ and iPCQ questionnaires will be filled in once pre- and a two times postoperatively by every patient. The OQUA will be filled in once preoperatively and three times postoperatively. The direct health-care costs will be recorded for the sponsor centre and extrapolated for the whole trial.

Nature and extent of the burden and risks associated with participation, benefit and group relatedness: The burden patients will experience by participating in this study will be filling in the questionnaire multiple times. The surgery, along with the imaging procedures (CT and DW-MRI), audiometry and follow-up appointments, is part of standard care and takes place irrespective of participation in this study.

1. INTRODUCTION AND RATIONALE

A cholesteatoma manifests through the retraction or ingrowth of the tympanic membrane's skin into the middle ear, thereby inducing chronic inflammation of the middle ear. It is diagnosed in the Netherlands approximately 1600 times per year. Due to the chronic nature of a cholesteatoma, this patient group presents frequently to the ENT outpatient clinic over a long period of time. Consequences of this pathology arise from the erosion and inflammation of surrounding structures resulting in a constellation of symptoms such as hearing loss, ear drainage and/or the presence of unresponsive granulation tissue. Additionally, patients may experience vertigo, tinnitus, earache, a feeling of pressure on/in the ear, change in taste, and an itch. These problems can have a severe impact, not only on physical health but are also associated with a negative effect on psychosocial wellbeing and the quality of life. The only effective treatment is the surgical removal of cholesteatoma. In the absence of surgical intervention, it can lead to severe complications, including meningitis, brain abscess, facial nerve paralysis, or permanent loss of auditory or vestibular function.

The goal of cholesteatoma treatment is to eradicate the disease and create a safe and dry ear, while preventing recurrence of pathology and preserving, or even optimizing, hearing. The best surgical approach has been subject of debate for decades. The two traditional approaches are: canal wall down (CWD) and canal wall up (CWU) tympanomastoidectomy. Although they both have their advantages and disadvantages, the CWU technique has increasingly become the standard approach. CWD surgery results in a radical cavity, which gives a wide visual exposure of the disease for removal and therefore minimizes the chance of leaving cholesteatoma behind. However, a radical cavity creates a situation that requires regular debridement, increased risk of recurrent infections, and makes the fitting of hearing aids more challenging. The CWU approach on the contrary, maintains the normal anatomy of the bony part of the ear canal. It results in less frequent inflammations, hearing aids fit better and the regular outpatient cleaning of the ear is not indicated (1,2). However, the major issue with the CWU treatment is the higher odds of recurrent and residual disease. Whereas CWD tympanomastoidectomy show recidivism - including both recurrent and residual disease - rates of 0 to 17%, CWU tympanomastoidectomy rates vary between 9% and 70% (3,4).

Over the past decade, there has been growing interest in finding ways to decrease the disease recurrence rates and improve post-operative hearing outcomes. One promising technique is the obliteration of the mastoid and epitympanic area after either CWD or CWU tympanomastoidectomy. Obliteration is the act of filling the cavity that is created to reach the middle ear with either bone dust (sometimes also referred to as bone pâté) or artificial material. The purpose is to create a situation in which the obliteration material prevents the tympanic membrane from retracting and therefore lowers the chance of cholesteatoma recurrence. Mercke was one of the first otologic surgeons to present promising outcomes after obliteration in combination with CWD tympanomastoidectomy (5).

When Mercke first presented his results, the so called ‘diffusion weighted magnetic resonance imaging’ (DW-MRI) was not yet developed, making the detection of cholesteatoma during follow-up more difficult. At this time, patients traditionally underwent a ‘second-look’ operation, meaning that after a certain period the ear was surgically opened again to check if the disease had come back. Surgeons that are opposed to or do not utilize the obliteration technique may be afraid that it might be more challenging to detect and remove a cholesteatoma that might have been left behind after obliteration. “The main limitation of the technique is the inability to explore the mastoid cavity during follow-up with the risk of developing a “silent cholesteatoma”, making radiological follow-up essential”(6). The development of the DW-MRI changed this somewhat by providing more diagnostic assurance and is currently used for the follow-up of patients that have been operated on. With the invention of the DW-MRI, the obliteration technique was therefore reintroduced and performed also subsequent to the CWU approach as patients could be followed more reliably and more otologists started believing in and using the obliteration technique. However, a limitation of radiological follow-up is the sensitivity of a DW-MRI, which ranges from 40-100% and the fact that cholesteatoma’s are only detected when they are larger than 3mm in size (7–16).

Recently, van der Toom et al. showed in their systematic review that mastoid obliteration seems to reduce recurrence rates for both the CWD and CWU approach when compared to CWD and CWU without obliteration (17). While they found recurrent and residual disease rates of 5.9% and 5.8%, respectively, for the CWD technique, the outcomes for CWU tympanomastoidectomy were better. For the latter technique, they presented a recurrent disease rate of 0.28% and a residual disease rate of 4.2%. Combining the CWU tympanomastoidectomy and obliteration technique therefore seems a good solution to preserve the normal anatomy of the ear canal, as well as minimize the chance of contracting recurrent or residual cholesteatoma.

Whether surgeons do or do not obliterate the mastoid cavity after CWU tympanomastoidectomy today, seems to depend mostly on their personal experience, preferences, and scientific beliefs. Within the present ENT community, the usefulness of the obliteration is still widely discussed and is still a topic of current interest in literature, during congresses and within ENT-societies. The superiority of the technique is merely explored retrospectively in the literature with several confounding factors and a high risk of bias (such as difference in but not limited to; patient-related factors, surgical technique; obliteration material; follow-up periods; exclusion/inclusion criteria). Furthermore, current research rarely categorizes outcomes based on cholesteatoma extension and localisation, as for example classified using the STAMCO stages (18).

In addition to recidivism of cholesteatoma, hearing is another important outcome measure. In the literature, most research is conducted on hearing outcomes after CWD or canal wall reconstruction surgery with mastoid and epitympanic obliteration. Numerous studies show that these two similar techniques preserve preoperative hearing outcomes, or even moderately improve it (19–23). However, there is limited literature available on hearing outcomes following CWU with obliteration. The few retrospective studies that have recently been carried out show promising results that this approach also ensures hearing preservation (24–26). When using the obliteration technique in the epitympanum (a part of the middle ear), a part of the ossicular chain (part of the malleus and incus) must be removed, which poses a risk for hearing loss after surgery. It is therefore useful to investigate the relation between CWU tympanomastoidectomy with obliteration and hearing outcomes to either confirm or deny the retrospective evidence that recurrence and residual rates are lower due to the obliteration.

Since the preferred technique of the otologic surgeon is merely supported by retrospective data, the need for high quality data to provide an answer to this debate is clear. Bringing change into practice is a difficult task that must be supported by high quality prospective research. The aim of this randomized controlled trial is therefore, firstly, to determine whether CWU tympanomastoidectomy with obliteration reduces the recurrence and residual rates of cholesteatoma compared to CWU tympanomastoidectomy without obliteration. Secondly, hearing outcomes will be evaluated to investigate whether one of the two mentioned techniques result in better post-operative hearing. Thirdly, the quality of life will be measured using a validated questionnaire (OQUA) and the cost-efficiency will be calculated.

2. OBJECTIVES

Primary Objective: The primary aim of this randomized controlled trial is to determine whether CWU tympanomastoidectomy with obliteration reduces the recurrence and residual rates of cholesteatoma compared to CWU tympanomastoidectomy without obliteration.

Secondary Objective(s):

- Hearing outcomes
- Health-related quality of life
- Describing localization and disease extension using the STAMCO classification
- Health economic cost analysis
- Comparing any differences in patient and surgical characteristics

3. STUDY DESIGN

The proposed study design is a multicenter two-armed randomized controlled trial. It will be conducted using a single blind method. The patients will not know which surgical approach they received, which is necessary for the subjective measurement of the quality of life questionnaire. The surgeon will know which procedure he/she has performed as this is unavoidable. The surgeon will not write down which procedure the patient received in the surgical report or in the letter to the general practitioner. It will only be recorded in Castor and in the RFS map. De blinding (single-sided) will only be lifted if it is in the interest of the safety of the participant. De participant and/or general practitioner can request this from the treating physician (ENT-surgeon) who performed the surgery. The ENT-surgeon will decide if lifting of the blinding is necessary and inform the researcher about this event. In case of emergency and in absence of the treating physician, other medical specialists can lift the blinding by checking the placement of the patient in the RFS map. All medical specialists have access to the RFS map with information of the participants where an inclusion table with randomization is stored. This way it is possible to lift blinding 24 hours a day. After completion of the last quality of life questionnaire (at 36 months) the treatment group will also be added to the patient file in HiX. The patients are randomly assigned to one of the two groups (A or B). A web-based randomization tool in Castor will be used for the allocation and will be accessed by the researcher. The study will be conducted with a sufficient duration to gather the sample size needed. The setting is multi-center; both secondary and tertiary university hospitals will participate. The estimated duration of the study is 7 years (84 months): 1 year will be allocated for completing the inclusions and follow-up will be completed in the following 5 years. The last year will be used to analyze the data and round up the trial.

All pre-operative measurements are part of standard care in the Netherlands and Belgium. Pre-operatively patients receive an audiometric exam, a physical examination of the ear, a CT-scan and fill in the patient-reported outcome measure (PROM). Follow-up will be planned at least at 6-18 weeks, at around 1 year and at around 3 years after surgery. It is important to note that conducting a CT scan and audiometry preoperatively as well as the DW-MRI's after 1 and 3 years are part of the standard care. In line with standard care, if there is a suspicion for recurrent or residual pathology (either clinically by micro-otoscopy or radiologically by DW-MRI) the treating physician will perform a revision surgery. If during revision surgery the suspicion is confirmed, the type of recidivism (residual or recurrent) is recorded and the follow-up of the patient ends. Data that is collected up until the point of detection of residual or recurrent disease will be used for analysis (this is <u>not</u> an exclusion criterium). If the suspicion is not confirmed (false-positive) then the patient will continue the follow-up as planned (unless a reconstructive procedure with the goal of improving hearing has been performed). During the three years of follow-up, patients should <u>not</u> undergo another type of ear surgery. If this is unavoidable, as judged by the treating physicians, the patient will be retrospectively excluded. In order to ensure that the same surgical data is collected in all participating centers, a standardized operation report will be written and shared by the sponsor (UMC Utrecht). This operation report will be recorded in the electronic patient dossier (with the exception of the information if obliteration was used or not) and transferred to Castor EDC. In Castor there will be a variable to also record if obliteration has taken place or not. The only methodology that is different to standard care is the randomization of the patients and the completion of the questionnaire. It is important to note that the technique used for the obliteration technique differs slightly between centers. The exact surgical technique will be described by the participating otologists and included in the description of the study, for example in a publication.

Figure 1. Flowchart of the study. *Standard care is indicated by **

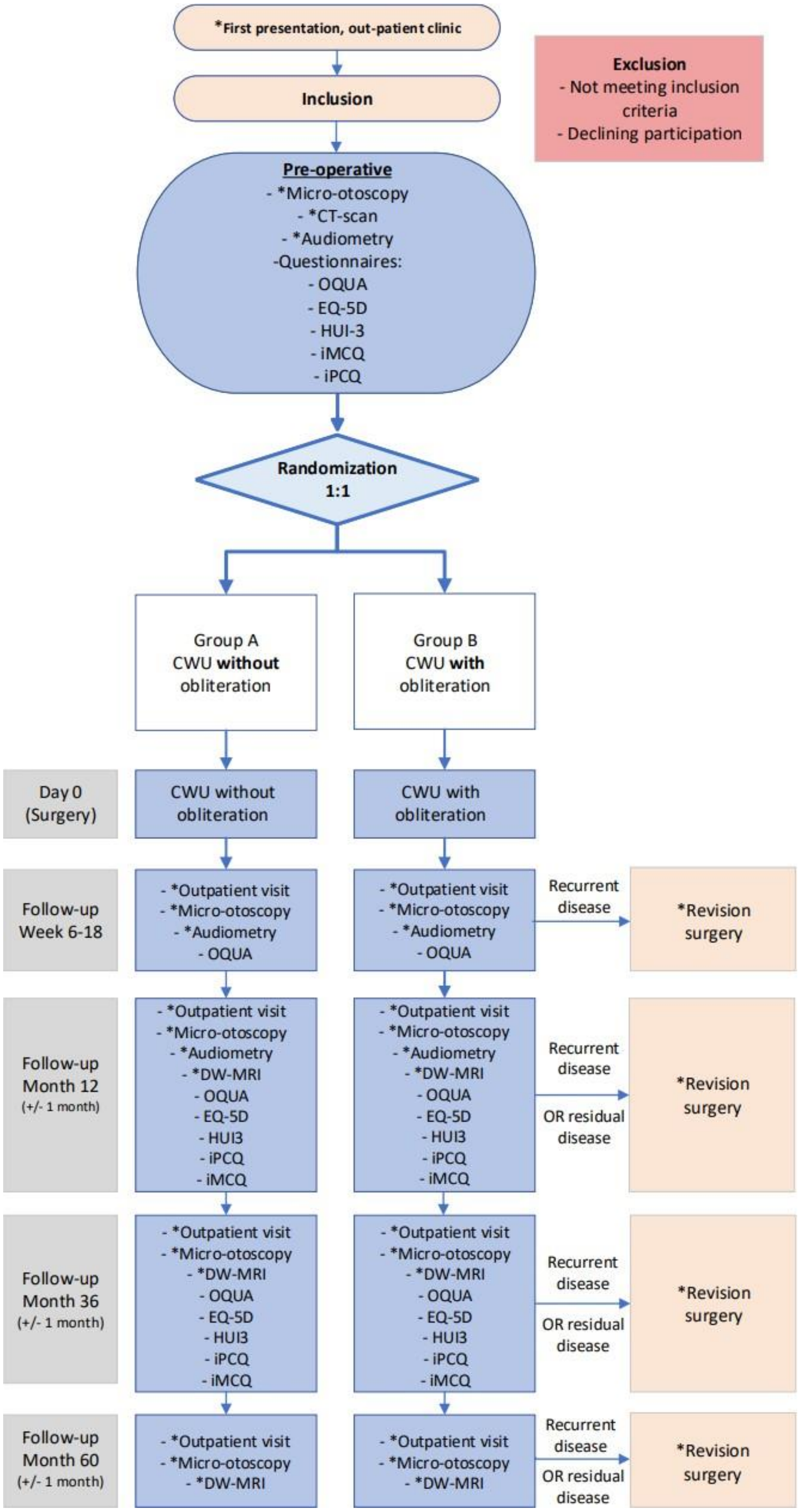

4. STUDY POPULATION

4.1 Population (base)

One-hundred thirty-two patients, above the age of 18, who are planned for surgical removal of a cholesteatoma using the CWU tympanomastoidectomy approach (with or without obliteration) between February 2025 and latest July 2026 in one of the participating centres will be asked to participate in this study. This includes all patients with an indication for a CWU tympanomastoidectomy, irrespective if this concerns a primary surgery or a revision surgery. We will not take into account gender, ethnic background or other characteristics of the study population as this has no influence on the disease or treatment. They must meet the following criteria to be eligible for the study.

4.2 Inclusion criteria

In order to be eligible to participate in this study, a subject must meet all of the following criteria:

- The patient is willing to participate and has provided written informed consent authorization before participating in the study
- The patient is ≥18 years of age at the time of consent
- The patient has sufficient understanding of the Dutch, French, or English written language
- The health status of the patient allows general anaesthesia and surgery for the removal of a cholesteatoma
- The patient is eligible to undergo a CWU tympanomastoidectomy approach based on the clinical and/or radiological suspicion for a cholesteatoma. Patients should only be included when the surgeon deems a transcanal approach not feasible and that, therefore, a mastoidectomy is necessary based on the pre-operative assessment.

4.3 Exclusion criteria

A potential subject who meets any of the following criteria will be excluded from participation in this study:

- Cases of revision surgery due to residual disease with a normal, intact or reconstructed tympanic membrane
- Cases where a previous obliteration has taken place
- Cases of congenital cholesteatoma
- Patient with an indication for the surgery due to a disease other than cholesteatoma (chronic otitis media)
- In rare cases patients may have a pathology which makes obliteration unavoidable due to the invasiveness of the disease (e.g. cholesteatoma extension into and near

total destruction of the bony ear canal or bony tegmen plate). This is judged by the treating physician. These patients cannot be included because randomisation is not a possibility in these cases.

- Severe comorbidity with an expected survival of less than five years
- Comorbidity or disorder which could interfere with the completion of questionnaires (e.g. known psychiatric disorder, mental retardation)
- Compromising anatomical situation (i.e. radical cavity, Congenital craniofacial anomalies with involvement of the temporal bone and including cleft palate)
- Contraindication to undergo a diffusion-weighted magnetic resonance imaging (DW-MRI) (e.g. claustrophobic, metal parts of implants in the body such as a pacemaker)

4.4 Sample size calculation

To detect a clinically relevant outcome of the primary objective (residual or recurrent disease) a sample size of at least 160 participants is needed. To calculate the sample size we used applicable and recent retrospective results of the UMC Utrecht (from January 2015 to March 2020) and reported recurrence rates from a systematic review of the literature, as this represents the most methodologically comparable and robust dataset currently available. We used our results to calculate the sample size needed with a power of 0.80. Relevant clinical difference was set at 20%. The calculation is based on a two independent proportions power analysis using a Z test with pooled variance with a alpha of 0.05 and a power of 0.80. Sample size calculations were performed with PASS 2008 and resulted in 80 patients per arm. To compensate for potential dropouts, a 10% margin is implemented, **resulting in 178 patients in total**.

Two Independent Proportions (Null Case) Power Analysis

Numeric Results of Tests Based on the Difference: P1 - P2
H0: P1-P2=0. H1: P1-P2=D1<>0. Test Statistic: Z test with pooled variance

Power	Sample Size Grp 1 N1	Sample Size Grp 2 N2	Prop|H1 Grp 1 or Trtmnt P1	Prop Grp 2 or Control P2	Diff if H0 D0	Diff if H1 D1	Target Alpha	Actual Alpha	Beta
0.8009	80	80	0.2000	0.4000	0.0000	-0.2000	0.0500	0.0472	0.1991

5. TREATMENT OF SUBJECTS

5.1 Investigational product/treatment

All subjects will undergo a canal wall up surgery to remove their cholesteatoma. Half of the cohort (group B) will undergo surgery *with* obliteration. Obliteration is to be performed using various materials: autologous bone dust, cartilage, muscle, synthetic material (Bonalive® or hydroxyapatite), or a combination of these. Only the outcomes of these two treatments (with or without obliteration) will be studied. No products will be studied.

6. METHODS

6.1 Study parameters/endpoints

6.1.1 Main study parameter/endpoint

The main endpoint is the number of patients with recurrent or residual cholesteatoma. Follow-up for recurrent/residual cholesteatoma will be performed after 6-18 weeks and after approximately 1, 3 and 5 years. Follow-up is done by micro-otoscopy and DW-MRI scans (after 1, 3 and 5 years) (see figure 1 for the follow-up steps). A newly formed retraction pocket from the tympanic membrane correlates with recurrent disease (clinical diagnosis), whereas cholesteatoma formation behind a normal or an intact tympanic membrane after primary surgery correlates with residual disease (radiologic diagnosis).

6.1.2 Secondary study parameters/endpoints

- **Baseline characteristics:**
    - Patient demographics (age (as collected by birthyear), gender), date of first presentation at the outpatient clinic, date of surgery, operated side (right/left), applied antibiotic treatment within in 2 weeks prior to surgery.
    - Co-morbidities and lifestyle factors: smoking, obesity, asthma, diabetes, COPD, allergies, rhinosinusitis.
    - Amount and type of all previous ear surgeries which are not mentioned in the exclusion criteria.
- **Hearing outcome:**
    - As measured by audiometric testing (pure-tone average thresholds (PTA) as calculated over 0.5, 1, 2 and 4 kHz for the air-conduction (AC) and bone-conduction (BC) and speech audiometry. This will be measured before the surgery, and at 6-18 weeks and around 1 year after surgery. The pre-operative audiogram should not have been measured more than 6 months prior to surgery.

- **Surgical characteristics:**
  - A short ‘standard’ surgical report/template written by the participating otologists per center will be collected and used in the scientific publication to demonstrate any potential differences in surgical technique. This **does not** include patient information.
  - STAMCO-classification: Localization and extension of the cholesteatoma, presence of preoperative complication status and the ossicular chain status before/at the time of removal of the cholesteatoma will be recorded. Any differences in outcomes in STAMCO classifications between the two randomized groups will be evaluated and described.

Figure 2: Localization, disease extension and complications as described by the STAMCO classification (18, ten Tije et al.)

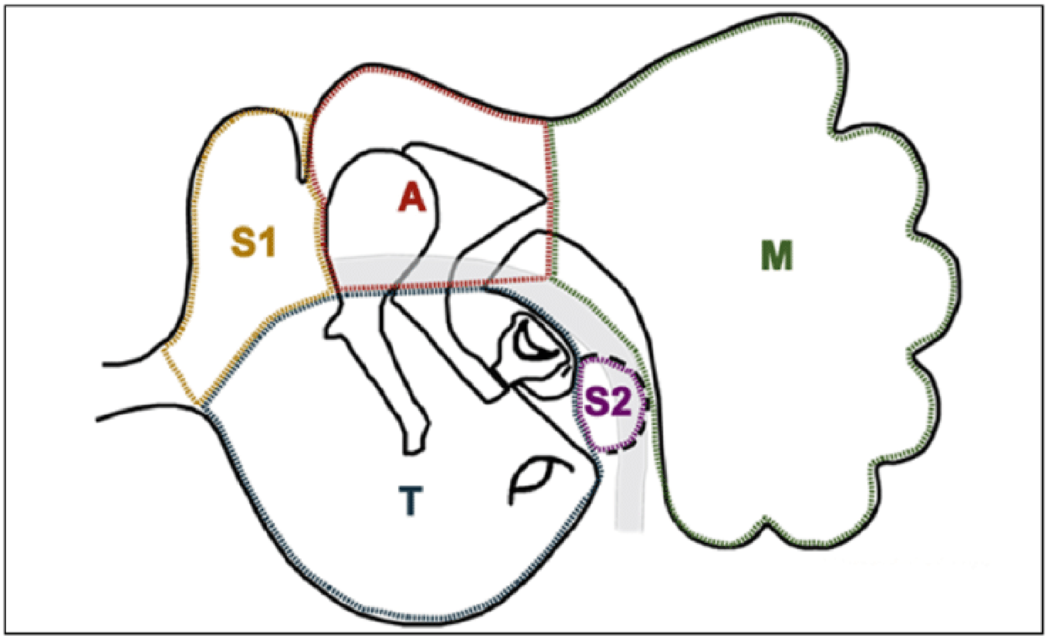

STAM	C	O
S1: Difficult anterior area	Cn: No complication	On: No ossicles missing or destructed
S2: Difficult posterior area	C1: Extracranial complication	O1: One ossicle missing or destructed, malleus present, stapes present, incus missing or (partially) destroyed
T: Tympanic cavity	C2: Intracranial complication	O2: Two ossicles missing or destroyed, malleus present, incus missing or partially destroyed, stapes suprastructure destroyed
A: Attic / epitympanic cavity		O3: Three ossicles missing or destroyed or any situation with a fixed footplate
M: Mastoid & Antrum		Ox: Unknown status of the ossicle

  - Material used for obliteration (Bonalive®, bonedust, hydroxyapatite, cartilage, muscle) as registered in the surgical report.

    - Ossicular chain reconstruction (OCR); performed (yes/no), if yes, the used material and type of reconstruction (PORP, TORP, use of incus interposition, type 3 tympanoplasty) will also be reported.
    - Surgical personnel: It is registered if an ENT-doctor in training (resident) or fellow has operated along with the specialist.
    - Surgical time: Surgery time is recorded in minutes for each surgery and described.
    - Revision surgery during follow-up: the information and observations as gathered by the surgical report will be collected from all revision surgeries performed during the follow-up of this study.
- **Complications**
    - Caused by the cholesteatoma (e.g. labyrinth fistula, dura defect, meningoencephalocele, dehiscence of facial nerve, interrupted bony tegmen plate, etc.).
    - Complications during or after surgery (e.g. wound infection, bleeding, damage to the inner ear (resulting in perceptive hearing loss or dizziness), damage to the facial nerve causing facial paralysis, damage to the taste nerve resulting in change in taste, intracranial complications (damage to the tegmen, liquor leakage), tinnitus).
    - Complications that occur within the first 6 weeks after surgery will be registered
- **Quality of life:** Is quality of life comparable between the two techniques? This will be measured using a validated patient reported outcome measure (PROM) in the form of an otological questionnaire (OQUA) and two generic quality of life questionnaires before and after the surgery (27).
    - **OQUA:** This is a otological questionnaire and includes 34 questions, covering eight major types of ear complaints (earache, pressure in the ear, hearing loss, tinnitus, otorrhoea, itch, dizziness, and loss of taste). It measures the frequency and severity of these ear complaints and the impact on quality of life.
    - **EQ5D-5L and HUI3:** The utility scores will be measured to be able to conduct an economic evaluation as described below.
- **Economic evaluation:** A cost-utility analysis will be performed according to the Dutch guideline for economic evaluations in healthcare. Differences with the Belgian guideline for economic evaluations, such the perspective, will be accounted for in the scenario analyses. For instance, in the base case, we will perform the analysis using a societal perspective. In the scenario analysis, we will include a healthcare perspective based on the Dutch base case analysis,

as well as a scenario analysis using healthcare perspective and Belgian unit costs and QoL value set. The cost-utility analysis will be performed with a lifetime time horizon. Data will be collected alongside the clinical trial and extrapolated over lifetime using a decision-analytical model. Unit costs of both procedures and of reinterventions will be calculated with a bottom-up approach using duration of surgery, personnel, disposables and materials used. Other healthcare resource use, for instance admissions, out-patient consultations, GP visits and paramedic care, will be collected using patient-reported healthcare resource use using the Institute for Medical Technology Assessment Medical Consumption Questionnaire (iMCQ). Productivity losses will be collected with the Institute for Medical Technology Assessment Productivity Cost Questionnaire (iPCQ). Both questionnaires will be adjusted for the patient population and the Belgian setting together with clinical experts and patient input. Healthcare resource use will be multiplied with unit costs from the Dutch costing manual and Belgian unit costs in a scenario analysis. The PIF is adjusted to enable validating healthcare resource use later on if necessary with claim data, such as in the IMA data linkage or VEKTIS data. For Belgian participants, the national number (RRN/INS) will be collected to enable possible data linkage with billing data from RIZIV/INAMI for validation purposes. If deemed necessary based on the trial results, approval will be requested from the competent chamber of the Information Security Committee to have the relevant study data linked with IMA data by a trusted third party (TTP, eHealth platform) using the patient national number. The national number will not be included in the trial database. This data linkage will be performed to obtain a more complete data set containing costs related to health care paid by the compulsory health insurance and the patient. This linked information will be used for the analysis of effectiveness and cost-effectiveness of the intervention.

Quality of life related to the procedures and (longer-term) hearing loss will be collected with HUI-3, specific for hearing problems. To validate use of HUI-3 in a cost-utility analyses, the EQ-5D-5L will be collected as well. Costs and quality of life will be calculated for each patient and for each health state in the decision-analytical model. Extensive sensitivity analyses will be performed, including deterministic (DSA), probabilistic sensitivity analysis (PSA), scenario analyses and VOI analyses, to estimate the impact of uncertainty on cost-effectiveness. The results of the cost effectiveness analysis will be combined with observed number eligible patients to estimate the budget impact.

6.2 Randomisation, blinding and treatment allocation

If they sign the informed consent form they will be randomly allocated to one of the two treatment groups (A or B) using the Randomization Tool in Castor. Patients will be randomly assigned to one of the two study groups (A or B). A website randomisation program built within Castor will be used for the randomization. After informed consent, patients will be randomized. This is a single blind study, meaning that only participants are blinded for the treatment allocation. The surgeons cannot be blinded, as they will know which procedure they performed. The surgeon will not write down which procedure the patient received in the surgical report or in the letter to the general practitioner. It will only be recorded in Castor and in the RFS map. De blinding (single-sided) will only be lifted if it is in the interest of the safety of the participant. De participant and/or general practitioner can request this from the treating physician (ENT-surgeon) who performed the surgery. The ENT-surgeon will decide if lifting of the blinding is necessary and inform the researcher about this event. Only in case of medical emergency and in absence of the treating physician, other medical specialists can lift the blinding by checking the placement of the patient in the RFS map. All medical specialists have access to the RFS map with information of the participants where an inclusion table with randomization is stored. This way it is possible to lift blinding 24 hours a day.

Stratification will be used to distribute the two groups as evenly as possible between the participating centres. Patients are randomized into one of two groups in an equal 1:1 allocation ratio. A 4,6,8 block randomization will be used.

6.3 Study procedures

The procedures and tests will be administered during the standard care process. The only extra procedures are the informed consent procedure, randomization process and the collection of questionnaires. The baseline characteristics, preoperative CT-scan, operative report, postoperative DW-MRI scan, and pre- and postoperative audiometric measurement will be collected and extracted from the electronic health record. The questionnaires and randomization will be performed and collected in Castor EDC. A clinical research file (CRF) is created to store all data, which is added to the database in Castor EDC (Amsterdam, the Netherlands). A research imaging archive (RIA) will be used to store the medical images which will be transferred (pseudonymized) to the sponsoring center.

Table 1. Study procedures

			**Follow-up (post-operative)**			
**Measurement**	**Pre-operative (baseline)**	**Surgery Day 0**	**6-18 weeks**	**12 months (+/- 1 month)**	**36 months (+/- 1 month)**	**60 months (+/- 1 month)**
CT-scan	X*			(X)*	(X)*	(X)*
Audiometry	X*		X*	X*		
Visit outpatient clinic and micro-otoscopy	X*		X*	X*	X*	X*
Inclusion	X					
Randomization	X					
Surgery		X*				
OQUA	X		X	X	X	
EQ-5D-5L	X			X	X	
HUI-3	X			X	X	
iMCQ	X			X	X	
iPCQ	X			X	X	
DW-MRI scan				X*	X*	X*
Registration of recurrent or residual disease (if applicable)			X*	X*	X*	X*
(x) = *depending on the necessity as judged by the treating physician, in line with best standard practice*						

6.4 Withdrawal of individual subjects

Subjects can leave the study at any time for any reason if they wish to do so without any consequences. The investigator can decide to withdraw a subject from the study for urgent medical reasons.

6.5 Replacement of individual subjects after withdrawal

See also section 4.4 sample size calculation. To anticipate possible withdrawal a 10% margin is taken into account to still provide sufficient power (of 0.80). An intention to treat analysis will be used, there is no reason to replace patients after withdrawal.

6.6 Follow-up of subjects withdrawn from treatment

During the study, each patient will stay in care of their own otorhinolaryngologist and audiologist. In case a patient is withdrawn from the study he/she will remain within regular medical care.

6.7 Premature termination of the study

In case of serious adverse events with higher frequency than reasonably might be expected and related to the mentioned surgical interventions, the research group will discuss further continuation of the study or possible pre-emptive termination.

6.8 Patient and public involvement (PPI)

This study will benefit from the expertise and input of patient representatives from relevant organizations in both participating countries: Hoormij (Netherlands) and Hoornetwerk (Belgium). Representatives will be consulted at predefined time points throughout the study, including during finalization of the study design, development of participant materials, and interpretation and dissemination of results. In addition, structured consultation meetings will be organized at least every six months to ensure ongoing input on study procedures and aspects relevant to patient experience. Support from both organizations has been confirmed, and letters documenting their willingness to contribute are included as an appendix to the protocol. An overview of the planned roles and timing of patient organization involvement across the different phases of the study is provided in a participationmatrix included in the appendix.

7. SAFETY REPORTING

7.1 Temporary halt for reasons of subject safety

In accordance to section 10, subsection 4, of the WMO, the sponsor will suspend the study if there is sufficient ground that continuation of the study will jeopardise subject health or safety. The sponsor will notify the accredited METC without undue delay of a

temporary halt including the reason for such an action. The study will be suspended pending a further positive decision by the accredited METC. The investigator will take care that all subjects are kept informed.

7.2 AEs, SAEs and SUSARs

7.2.1 Adverse events (AEs)

Adverse events are defined as an undesirable experience occurring to a subject during the study. Adverse events that will be reported are the following: Complications during or after surgery (e.g. wound infection, post-operative bleeding, damage to the taste nerve resulting in change in taste, tinnitus). These are the same complications that are also mentioned by the treating physician in standard clinical care and for which informed consent is asked when discussing the surgical operation. From professional experience we will report complications that occur within the first 6 weeks after surgery. We consider this to be a relevant period in which complications due to the intervention can occur.

7.2.2 Serious adverse events (SAEs)

A serious adverse event is any untoward medical occurrence or effect that

- results in death;
- is life threatening (at the time of the event);
- requires hospitalisation or prolongation of existing inpatients' hospitalisation;
- results in persistent or significant disability or incapacity; or
- any other important medical event that did not result in any of the outcomes listed above due to medical or surgical intervention but could have been based upon appropriate judgement by the investigator.

An elective hospital admission will **not** be considered as a serious adverse event.

This study is expected to pose a negligible risk for patients; any SAEs occurring during the study period are expected to be related to the standard treatment and not to the study intervention. SAE's which could occur as a result of the operation and are always discussed with the patient beforehand as part of standard care are: damage to the inner ear (resulting in perceptive hearing loss or dizziness), damage to the facial nerve causing facial paralysis, intracranial complications. These will only be reported if they occur during or within the first six weeks after surgery as a later onset cannot be seen as a consequence of the intervention.

The investigator will report all SAEs to the sponsor without undue delay after obtaining knowledge of the events. The investigator reports this to the principal investigator and coordinating investigator of the sponsor (UMC Utrecht) per secured e-mail. The sponsor

will report the SAEs through the web portal *ToetsingOnline* to the accredited METC that approved the protocol, within 7 days of first knowledge for SAEs that result in death or are life threatening followed by a period of maximum of 8 days to complete the initial preliminary report. All other SAEs will be reported within a period of maximum 15 days after the sponsor has first knowledge of the serious adverse events.

7.3 Follow-up of adverse events

From professional experience we will report complications that occur within the first 6 weeks after surgery. We consider this to be a relevant period in which complications due to the intervention can occur. All AEs will be followed until they have abated, or until a stable situation has been reached. Depending on the event, follow up may require additional tests or medical procedures as indicated, and/or referral to the general physician or a medical specialist. SAEs need to be reported till end of study within the Netherlands, as defined in the protocol.

8. STATISTICAL ANALYSIS

Data will be collected in Castor EDC and exported for Statistical analysis in IBM SPSS Data will be collected in Castor EDC and exported for Statistical analysis in IBM SPSS Statistics 29.0 (SPSS Inc., Chicago, IL, USA). Data will be presented according to the Consolidated Standards of Reporting Trials (CONSORT) Statement, an international guideline on adequate reporting of RCTs. To assess whether continuous variables are normally distributed, histograms and the Kolmogorov-Smirnoff test will be computed. Continues data will be expressed as mean ± standard deviation (SD) when normally distributed, and as median ± interquartile range (IQR) when skewed. Number of cases and percentages will be presented for categorical variables. A p-value of less than 0.05 will be regarded as statistically significant for all calculations.

8.1 Primary study parameter(s)

The data of our primary outcome are quantitative and will be presented as frequencies and proportions. To compare the difference in recidivism (recurrent and residual disease) at 12, 36 and 60 months between both surgical groups the Chi-squared test or Fisher's exact test will be used. The relative risk (RR) and absolute risk difference (RD) will be calculated as effect sizes using a generalized linear model for binominal outcome with a log-link (for RR) and identity link (RD). The primary analysis will be adjusted for site by including centre as covariate in a logistic regression model with treatment arm as the main predictor. All effect sizes will be accompanied by 95% confidence intervals. To graphically demonstrate the rates of recidivism, recurrent and residual disease over the follow-up time, a Kaplan-Meier analysis will also be performed and survival curves will be constructed. Multiple imputation will be used to deal with missing

outcomes. A 20% difference is defined as the threshold for clinical relevance and will be consistently applied in both clinical effectiveness analyses and the health economic evaluation.

8.2 Secondary study parameter(s)

Differences in STAMCO

- Within the STAMCO classification groups (STAM 1,2,3) the Chi-square test will be used to assess the differences in recidivism.

  Hearing outcome

- For each patient the mean difference of the four frequencies (500,1000,2000,4000 Hz) between pre- and postoperative audiograms will be calculated to yield the air-bone gap. Hearing outcomes will be compared between the treatment arms using a mixed model for repeated measures (MMRM) with fixed effects for treatment arm and timepoint (6-18 weeks and 12 months) and their two-way interaction and a random effect for subject. Analyses will be adjusted for the hearing outcome at baseline. Primary inference for hearing outcomes will be based on the estimated mean difference at 12 months. If assumptions of normality of residuals is not met, transformations of the outcome will be considered or alternatively non-parametric tests Mann-Whitney U test will be used to compare the audiological data between the two groups (A,B) separately at 6-18 weeks and 12 months. Hearing results will be visualized using the Amsterdam Hearing Evaluation Plot (AHEP) (28). To illustrate the outcomes of the speech audiograms, a scatterplot will be made according to Stanford medicine. https://hearingoutcomes.stanford.edu/

PROM (OQUA)

- The OQUA is a validated otological questionnaire and includes 34 questions, covering eight major types of ear complaints (earache, pressure in the ear, hearing loss, tinnitus, otorrhoea, itch, dizziness, and loss of taste). It measures the frequency and severity of these ear complaints and the impact on quality of life. The frequency of the ear complaints is expressed on a scale from 1-5, whereas the impact on quality of life is answered on a scale from 0-100. OQUA will be compared between treatment arms using a mixed model for repeated measures (MMRM) with fixed effects for treatment arm and timepoint (12 months, 36 months) and their two-way interaction. Analyses will be adjusted for OQUA at baseline. Primary inference will be based on the estimated mean difference at 36 months.

Baseline variables (patient characteristics, complications, surgical characteristics)

- Will be described using simple descriptive statistics using means/median and standard

deviations/interquartile range for the continuous variables, whereas categorical variables are expressed as absolute and relative frequencies. Continuous variables will be tested for normality using the Kolmogorov-Smirnov test and by computing histograms and consequently expressed as mean ± standard deviation (SD) when normally distributed, and as median ± interquartile range (IQR) when skewed.

Economic evaluation

- A cost utility analysis will be performed as described under the methods section.

The amount of missing data will be mentioned in the results. Missing data will be imputed using multiple imputation. All analyses will be performed on an intention-to-treat basis. Additionally, dropout rates will be monitored, and any differential attrition between treatment arms will be appropriately accounted for in the statistical analysis.

8.3 Interim analysis (if applicable)

Interim analysis is not deemed necessary, as the study population is not exposed to a considerable increased risk or experimental jeopardizing exposure.

9. ETHICAL CONSIDERATIONS

9.1 Regulation statement

The study will be conducted according to the principles of the Declaration of Helsinki (version 2024, https://www.wma.net/policies-post/wma-declaration-of-helsinki/) and in accordance with the Medical Research Involving Human Subjects Act (WMO). The Belgian Center (UZ Leuven) will comply to their own local rules and regulations which may or may not differ from those stated by the WMO.

9.2 Recruitment and consent

Screening for in- and exclusion criteria will be performed by the treating physician. Potentially eligible patients who will undergo a canal wall up surgery for a cholesteatoma will be informed about the study by the treating physician. The treating physician will provide oral and written information (in the form of the information letter including informed consent form) about the study and can when necessary give answers to content-related questions (for example about the surgery). The treating physician will ask the patient if their contact details (name, e-mail address and phone number) may be shared with the study-team member. If the patient gives consent, the treating physician will provide the study-team member with the contact details of the patient.

A trained study-team member will contact the patient for informed consent after a reasonable consideration of at least one day or as much time as needed. The patient has the opportunity to ask questions during this period and during this contact moment. Once all questions have been satisfactorily answered, the patient will be asked to sign the consent form previously given to them by the treating physician or study-team member and return it per post to the department of ENT-surgery of the local hospital (with a return envelope). The trained study-team member will sign the informed consent form as well and return one copy to the patient and keep the original informed consent form for administration. The original informed consent form is stored in a designated folder in a secure location at the local hospital.

After informed consent, the patient will be included in the study and assigned a study number by the researcher and randomly allocated to either group A or B. Participation will be registered in the electronic patient dossier. If the patient does not want to participate, contact with the study-member (not the treating physician) will be terminated. If the patient decides at a later point, after signing the informed consent form or during the study, that they want to stop participation, then the at that point collected data will not be deleted as this would introduce a significant bias into our database. It will also be recorded when patients have declined participation by the treating physician to avoid that the patient is approached again for participation or questions.

9.3 Benefits and risks assessment, group relatedness

The canal wall up procedure with or without obliteration is part of the (inter)national standard care of patients with cholesteatoma. The choice of technique depends largely on the preference and experience of the surgeon. In the literature the obliteration technique is described to result in good hygienic outcomes, the benefit is however not clearly defined. The participating patients in this study are not exposed to any additional risks or harms due to these interventions. Therefore the risk of this research can be considered negligible.

9.4 Compensation for injury

The sponsor/investigator has a liability insurance which is in accordance with article 7 of the WMO. The sponsor (also) has an insurance which is in accordance with the legal requirements in the Netherlands (Article 7 WMO). This insurance provides cover for damage to research subjects through injury or death caused by the study. The insurance applies to the damage that becomes apparent during the study or within 4 years after the end of the study.

10. ADMINISTRATIVE ASPECTS, MONITORING AND PUBLICATION

10.1 Handling and storage of data and documents

Data handling and protection is conducted according to applicable laws and regulations (i.e. GCP, 21 CFR Part 11, GDPR, and ISO 27001, 9001 compliant standards). Confidentiality will be maintained at all times, participant information will not be disclosed to third parties. Once the informed consent is signed by both parties according to the steps described in 9.2, each patient will receive a unique study number to protect their privacy. The original informed consent form will be stored at the local department in a lockable cupboard and a copy will be added to the research file of the patient in Castor. The key to the patient's personal data (name and patient ID) will be safeguarded by the investigator, who is the only one with access to the source data. Every centrum will register their own results in Castor EDC with access control, so that only persons specifically mentioned in the informed consent form have access to the data. All data will be collected using an electronic data capture tool (Castor EDC, a GCP-compliant database). Castor EDC features build in data trails, skips and validation checks. The healthcare data of patient related specifications, CT and DW-MRI images, and any other data as specified in section 6 (Methods) will be derived from the electronic health record. Radiographic scans from PACS are made available for research via our Research Imaging Architecture (RIA). The clinical database will be supplemented with the outcome of a validated questionnaire (the OQUA questionnaire, please see paragraph 3) which will be automatically sent via an email url-link via Castor at set times. The e-mail address (used for questionnaires) will be entered into Castor in an encrypted way and only retraceable to the centre of origin of the patient. Only the team of investigators will have access to the database files in Castor. All generated (meta)data will be stored in a secure research folder structure for access control in each separate participating centre, with only pseudonymized data leaving their centre of origin. To be able to reproduce the study findings and to help future users to understand and reuse the data, all changes made to the raw data and all steps taken in the analysis will be documented in text document. The database file will be kept for 15 years after the study has ended. More details can be found in the Data Management Plan (https://dmponline.dcc.ac.uk/plans/142594).

10.2 Monitoring and Quality Assurance

We deem the risk classification as negligible as there is only a small risk of damage for the patient as the methodology of this study is also part of the standard medical care. All centers will have one initiation visit, four monitoring visits and one close-out visit (six in total for the full study duration). The visits of the sponsor will be on-site. For the regular monitoring visits of the participating centers see K6. Monitoring plan. Monitoring will take

be done by a qualified monitor working at Julius Clinical for the Dutch participating centres, for optimal quality assurance according to the NFU guidelines. The monitoring for the Belgian centres will be performed by the Clinical Trial Centre (CTC) of UZ Leuven, which will mirror the monitor plan as performed by Julius Clinical to ensure comparable monitoring procedures across centres in both countries. Depending on the findings during the study, on-site visits can also be conducted at the other participating centres if necessary. For detailed information about our monitoring plan see file K6.

10.3 Amendments

Amendments are changes made to the research after a favourable opinion by the accredited METC has been given. All amendments will be notified to the METC that gave a favourable opinion. All substantial amendments will be notified to the METC and to the competent authority. Non-substantial amendments will not be notified to the accredited METC and the competent authority, but will be recorded and filed by the sponsor.

10.4 Annual progress report

The sponsor/investigator will submit a summary of the progress of the trial to the accredited METC once a year. Information will be provided on the date of inclusion of the first subject, numbers of subjects included and numbers of subjects that have completed the trial, serious adverse events/ serious adverse reactions, other problems, and amendments.

10.5 Temporary halt and (prematurely) end of study report

The investigator/sponsor will notify the accredited METC of the end of the study within a period of 8 weeks. The end of the study is defined as the last patient's last visit. The sponsor will notify the METC immediately of a temporary halt of the study, including the reason of such an action. In case the study is ended prematurely, the sponsor will notify the accredited METC within 15 days, including the reasons for the premature termination. Within one year after the end of the study, the investigator/sponsor will submit a final study report with the results of the study, including any publications/abstracts of the study, to the accredited METC.

10.6 Public disclosure and publication policy

The results (positive or negative) of this study will be disclosed unreservedly. Data and results of research are owned by the investigators. The results of research will be submitted for publication to peer-reviewed, open access scientific journals. Disputes on the interpretation of the results may not lead to an unnecessary delay in publication. None of the parties concerned has a right of veto. The parties concerned will attempt to resolve

disputes by negotiation. Should one of the parties feel that it has been disadvantaged, or should any other problem relating to publication arise, the parties will contact the medical ethics committee for mediation.

11. REFERENCES

1. Tos M, Lau T. Late Results of Surgery in Different Cholesteatoma Types. ORL. 1989;51(1):33–49.
2. Nikolopoulos TP, Gerbesiotis P. Surgical management of cholesteatoma: The two main options and the third way – atticotomy/limited mastoidectomy. Int J Pediatr Otorhinolaryngol. 2009 Sep;73(9):1222–7.
3. Kerckhoffs KGP, Kommer MBJ, van Strien THL, Visscher SJA, Bruijnzeel H, Smit AL, et al. The disease recurrence rate after the canal wall up or canal wall down technique in adults. Laryngoscope. 2016 Apr 25;126(4):980–7.
4. Tomlin J, Chang D, McCutcheon B, Harris J. Surgical Technique and Recurrence in Cholesteatoma: A Meta-Analysis. Audiology and Neurotology. 2013;18(3):135–42.
5. Mercke U. The cholesteatomatous ear one year after surgery with obliteration technique. Am J Otol. 1987 Nov;8(6):534–6.
6. Bovi C, Luchena A, Bivona R, Borsetto D, Creber N, Danesi G. Recurrence in cholesteatoma surgery: what have we learnt and where are we going? A narrative review. Acta Otorhinolaryngologica Italica. 2023 Apr;43(2 (Suppl. 1)):S48–55.
7. Lingam RK, Bassett P. A Meta-Analysis on the Diagnostic Performance of Non-Echoplanar Diffusion-Weighted Imaging in Detecting Middle Ear Cholesteatoma: 10 Years On. Otology & Neurotology. 2017 Apr;38(4):521–8.
8. Khemani S, Lingam RK, Kalan A, Singh A. The value of non-echo planar HASTE diffusion-weighted MR imaging in the detection, localisation and prediction of extent of postoperative cholesteatoma. Clinical Otolaryngology. 2011 Aug;36(4):306–12.
9. Ilica AT, Hidir Y, Bulakbasi N, Satar B, Guvenc I, Arslan HH, et al. HASTE diffusion-weighted mri: reliability for detection of cholesteatoma. Diagnostic and Interventional Radiology. 2011;
10. Huins CT, Singh A, Lingam RK, Kalan A. Detecting cholesteatoma with non-echo planar (HASTE) diffusion-weighted magnetic resonance imaging. Otolaryngology–Head and Neck Surgery. 2010 Jul;143(1):141–6.
11. Horn RJ, Gratama JWC, van der Zaag-Loonen HJ, Droogh-de Greve KE, van Benthem PPG. Negative Predictive Value of Non-Echo-Planar Diffusion Weighted MR Imaging for the Detection of Residual Cholesteatoma Done at 9 Months After Primary Surgery Is not High Enough to Omit Second Look Surgery. Otology & Neurotology. 2019 Aug;40(7):911–9.
12. Garrido L, Cenjor C, Montoya J, Alonso A, Granell J, Gutiérrez-Fonseca R. Capacidad diagnóstica de la resonancia magnética con técnica de difusión no eco-planar en la detección de colesteatomas primarios y recurrentes. Acta Otorrinolaringol Esp. 2015 Jul;66(4):199–204.
13. Foti G, Beltramello A, Minerva G, Catania M, Guerriero M, Albanese S, et al. Identification of residual–recurrent cholesteatoma in operated ears: diagnostic accuracy of dual-energy CT and MRI. Radiol Med. 2019 Jun 2;124(6):478–86.

14. De Foer B, Vercruysse JP, Bernaerts A, Deckers F, Pouillon M, Somers T, et al. Detection of Postoperative Residual Cholesteatoma With Non-Echo-Planar Diffusion-Weighted Magnetic Resonance Imaging. Otology & Neurotology. 2008 Jun;29(4):513–7.
15. Bakaj T, Zbrozkova LB, Salzman R, Tedla M, Starek I. Recidivous cholesteatoma: DWI MR after canal wall up and canal wall down mastoidectomy. Bratisl Lek Listy. 2016;117(9):515–20.
16. Allam HS, Abdel Razek AAK, Ashraf B, Khalek M. Reliability of diffusion-weighted magnetic resonance imaging in differentiation of recurrent cholesteatoma and granulation tissue after intact canal wall mastoidectomy. J Laryngol Otol. 2019 Dec 18;133(12):1083–6.
17. van der Toom HFE, van der Schroeff MP, Pauw RJ. Single-Stage Mastoid Obliteration in Cholesteatoma Surgery and Recurrent and Residual Disease Rates. JAMA Otolaryngology–Head & Neck Surgery. 2018 May 1;144(5):440.
18. ten Tije FA, Merkus P, Buwalda J, Blom HM, Kramer SE, Pauw RJ, et al. Practical applicability of the STAMCO and ChOLE classification in cholesteatoma care. European Archives of Oto-Rhino-Laryngology. 2021 Oct 18;278(10):3777–87.
19. Lee H, Chao J, Yeon Y, Kumar V, Park C, Kim H. Canal reconstruction and mastoid obliteration using floating cartilages and musculoperiosteal flaps. Laryngoscope. 2017;
20. Tos M, Lau T. Late Results of Surgery in Different Cholesteatoma Types. ORL. 1989;51(1):33–49.
21. Vercruysse JP, van Dinther JJS, De Foer B, Casselman J, Somers T, Zarowski A, et al. Long-term Results of Troublesome CWD Cavity Reconstruction by Mastoid and Epitympanic Bony Obliteration (CWR-BOT) in Adults. Otology & Neurotology. 2016 Jul;37(6):698–703.
22. Weiss NM, Bächinger D, Botzen J, Großmann W, Mlynski R. Mastoid cavity obliteration leads to a clinically significant improvement in health-related quality of life. European Archives of Oto-Rhino-Laryngology. 2020 Jun 6;277(6):1637–43.
23. Roux A, Bakhos D, Lescanne E, Cottier JP, Robier A. Canal wall reconstruction in cholesteatoma surgeries: rate of residual. European Archives of Oto-Rhino-Laryngology. 2015 Oct 17;272(10):2791–7.
24. Westerberg J, Mäki-Torkko E, Harder H. The evaluation of canal wall up cholesteatoma surgery with the Glasgow Benefit Inventory. European Archives of Oto-Rhino-Laryngology. 2020 Jan 4;277(1):61–8.
25. Westerberg J, Mäki-Torkko E, Harder H. Cholesteatoma surgery with the canal wall up technique combined with mastoid obliteration: results from primary surgery in 230 consecutive cases. Acta Otolaryngol. 2018 May 4;138(5):452–7.
26. Hellingman CA, Geerse S, de Wolf MJF, Ebbens FA, van Spronsen E. Canal wall up surgery with mastoid and epitympanic obliteration in acquired cholesteatoma. Laryngoscope. 2019 Apr 8;129(4):981–5.
27. Kraak JT, van Dam TF, van Leeuwen LM, Kramer SE, Merkus P. The Otology Questionnaire Amsterdam: A generic patient-reported outcome measure about the severity and impact of ear complaints. Validation, reliability and responsiveness. Clinical Otolaryngology. 2020 Jul 8;45(4):506–16.
28. Bruijn, A.J.G. & Tange, Rinze & Dreschler, Wouter. (2002). The Amsterdam hearing evaluation plots (AHEP's): A method of reporting individual hearing results. 8. 7-10.
